# Antitumor Activity and Mechanism of Action of the Antimicrobial Peptide AMP-17 on Human Leukemia K562 Cells

**DOI:** 10.3390/molecules27228109

**Published:** 2022-11-21

**Authors:** Zhuqing Tian, Longbing Yang, Mingjiao Huang, Chaoqin Sun, Mingming Chen, Wenjing Zhao, Jian Peng, Guo Guo

**Affiliations:** 1 The Key and Characteristic Laboratory of Modern Pathogen Biology, School of Basic Medical Sciences, Guizhou Medical University, Guiyang 550025, China; 2 Key Laboratory of Environmental Pollution Monitoring and Disease Control, Ministry of Education, Guizhou Medical University, Guiyang 550025, China; 3 Department of Clinical Laboratory, Guiyang Public Health Clinical Center, Guiyang 550004, China; 4 Translational Medicine Research Center, Guizhou Medical University, Guiyang 550025, China

**Keywords:** antimicrobial peptides, AMP-17, human leukemia K562 cells, anti-tumor, membrane-disruption, apoptosis

## Abstract

Cancer is one of the most common malignant diseases in the world. Hence, there is an urgent need to search for novel drugs with antitumor activity against cancer cells. AMP-17, a natural antimicrobial peptide derived from *Musca domestica*, has antimicrobial activity against Gram-positive bacteria, Gram-negative bacteria, and fungi. However, its antitumor activity and potential mechanism of action in cancer cells remain unclear. In this study, we focused on evaluating the in vitro antitumor activity and mechanism of AMP-17 on leukemic K562 cells. The results showed that AMP-17 exhibited anti-proliferative activity on K562 cells with an IC_50_ value of 58.91 ± 3.57 μg/mL. The membrane integrity of K562 was disrupted and membrane permeability was increased after AMP-17 action. Further observation using SEM and TEM images showed that the cell structure of AMP-17-treated cells was disrupted, with depressions and pore-like breaks on the cell surface, and vacuolated vesicles in the cytoplasm. Furthermore, further mechanistic studies indicated that AMP-17 induced excessive production of reactive oxygen species and calcium ions release in K562 cells, which led to disturbance of mitochondrial membrane potential and blocked ATP synthesis, followed by activation of Caspase-3 to induce apoptosis. In conclusion, these results suggest that the antitumor activity of AMP-17 may be achieved by disrupting cell structure and inducing apoptosis. Therefore, AMP-17 is expected to be a novel potential agent candidate for leukemia treatment.

## 1. Introduction

Despite the remarkable research advances in cancer treatment in recent years, cancer remains a significant cause of morbidity and mortality globally [1,2]. As a result, cancer has become one of the major diseases that seriously threaten human health. It is estimated that there are approximately 19.3 million new cancer cases and 10 million cancer deaths worldwide, with leukemia accounting for 2.7% of new cases and 3.1% of mortality [2]. Leukemia is a class of malignant clonal diseases that involve blood and hematopoietic stem cells, with a high mortality rate of malignancies in children and adults [2,3]. In 2022, 60,700 leukemia cases and 24,000 cases deaths are projected to occur in the United States [4]. Therefore, there is an urgent need to develop a novel and effective anti-cancer drug and innovative therapy.

At present, chemotherapy remains the treatment of choice for leukemia, but its long-term use can lead to drug resistance and even multidrug resistance (MDR) in patients during therapy [5,6]. In addition, resistance of leukemic cells to chemotherapeutic agents is a major factor causing treatment failure and disease relapse [7]. Molecularly targeted drugs have been reported to act specifically on leukemic cells, but long-term use can also cause drug resistance and even side effects such as arterial occlusion and venous thrombosis [8,9]. Thus, the successful treatment of this disease remains one of the most challenging and problematic types of cancer.

Antimicrobial peptides (AMPs), a class of peptide-like active substances produced in the innate immune system of organisms to defend against invasion by external pathogens, are widely present in a variety of organisms [10]. Although AMPs are known for killing microbes, there are increasing reports that AMPs exhibit anticancer properties [11,12]. Currently, AMPs have been shown to be highly efficient in antitumor; however, their antitumor mechanisms have not been fully elucidated [13]. It has been found that many AMPs of natural origin inhibit cancer cells mainly through membranolytic or non-membranolytic mechanisms [14]. For example, PNW [15], Polybia-MP1 [16], and Pardaxin [17] kill cancer cells by disrupting the cell membrane. Non-membranolytic mechanisms are mainly involved in ROS accumulation, DNA damage, mitochondrial membrane potential depolarization, autophagy, and apoptosis [18,19]. Pardaxin, an antimicrobial peptide isolated from secretions of the fish, the Red Sea Moses sole, triggered caspase-dependent, and ROS-mediated apoptosis in HT-1080 cells [18]. In addition, FK-16 derived from LL-37 induced caspase-independent apoptosis and autophagy through the p53-Bcl-2/Bax cascade reaction common in colon cancer cells [20]. Therefore, AMPs may be potential candidates for future cancer therapy.

Antimicrobial peptide-17 (AMP-17) is a naturally antimicrobial peptide derived from *Musca domestica* and consists of 164 amino acids [21]. In previous studies, AMP-17 has been shown to exhibit antimicrobial activity against Gram-negative bacteria, Gram-positive bacteria, and pathogenic yeasts, and is not significantly toxic to normal human erythrocytes [21,22,23]. However, it remains unclear whether AMP-17 has antitumor activity and through which pathways it exerts its antitumor effects. In this study, we reported that AMP-17 has antitumor activity on chronic myeloid leukemia cells K562 in vitro and characterized its mechanism of action on K562 cells as possibly related to membrane disruption and apoptosis. Therefore, the results of the present study provide further confirmation of the possibility of AMP-17 as a promising anticancer drug and provide some experimental basis for exploring its broader pharmacological effects.

## 2. Results

### 2.1. AMP-17 Exhibits Antiproliferative Activity on K562 Cells

**Figure 1:**
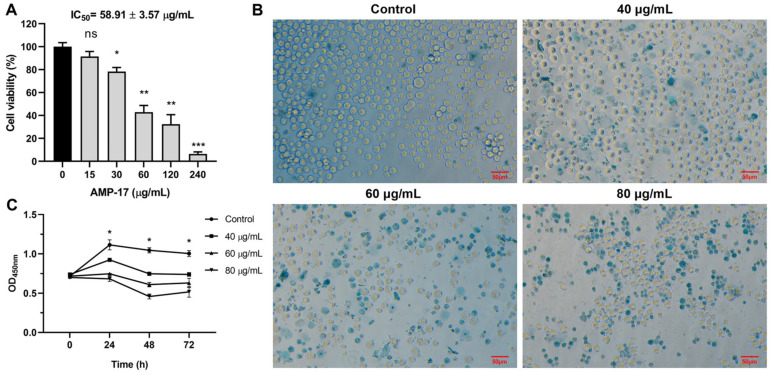
AMP-17 showed antitumor activity against K562 cells. (**A**) Cell viability of AMP-17 against K562 cells was determined using the CCK-8 assay (* *p* < 0.05, ** *p* < 0.01, *** *p* < 0.001, ns: not significant). The assay was performed in triplicate and repeated three times. (**B**) Trypan blue stained images of K562 cells treated with or without AMP-17. Images were captured using a 40× power field. Scale bar, 50 μm. (**C**) Cell proliferation of K562 cells treated with AMP-17 (40, 60 and 80 μg/mL) for 24, 48, and 72 h was determined using the CCK-8 assay. (* *p* < 0.05). The assay was repeated three times independently.

To determine the antitumor activity of AMP-17 on K562 cells, cell viability was determined using the CCK-8 assay. The results showed that AMP-17 was able to inhibit the cell proliferation of K562 cells in a dose-dependent manner with an IC_50_ value of 58.91 ± 3.57 μg/mL (Figure 1A). Subsequently, we further observed the morphology and survival of cells treated or untreated with AMP-17 by Trypan blue staining. The results showed that the untreated cells had normal morphology and a good glossy appearance, while the morphology of cells treated with AMP-17 was crinkled and the number of deaths increased (Figure 1B).

Next, we dynamically monitored the proliferation effect of AMP-17 on K562 cells using time-growth curves. As shown in Figure 1C, the cells in the untreated group proliferated rapidly within the monitoring range of 0–72 h, whereas the proliferation rate of the cells decreased significantly after AMP-17 treatment (*p* < 0.05), suggesting that AMP-17 has an anti-proliferative effect on K562 cells.

### 2.2. AMP-17 Induces Membrane Disruption of K562 Cells

**Figure 2:**
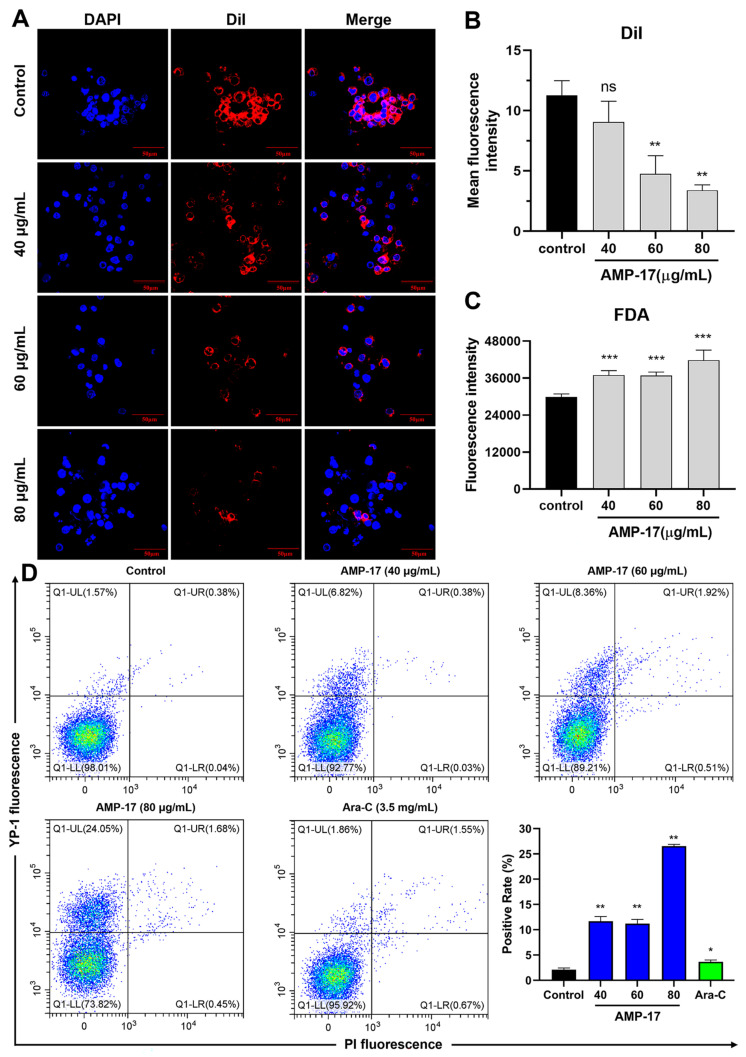
Effect of AMP-17 on the cell membrane of K562 cells. (**A**) Membrane disruption in AMP-17-treated K562 cells was observed using DiI dye and visualized using confocal laser confocal microscopy (CLSM). Images were captured using a 60× power field. Scale bar, 50 μm. (**B**) Histogram showing the mean fluorescence intensity of DiI in K562 cells treated with or without AMP-17 (** *p* < 0.01). (**C**) Fluorescence intensity of intracellular FDA efflux from K562 cells treated with different concentrations of AMP-17 (40, 60 and 80 μg/mL) for 24 h (*** *p* < 0.001). The experiment was performed in triplicate and repeated twice. (**D**) Membrane permeability of AMP-17-treated K562 cells was detected by double staining with YP-1/PI and analyzed using flow cytometry. Ara-C was used as a drug control (* *p* < 0.05, ** *p* < 0.01). The experiment was independently repeated three times.

To elucidate the membrane-related mechanisms of AMP-17, this study explored several aspects. To begin with, we observed the effect of AMP-17 on the cell membrane structure using Dil dye. The results showed that the cell membrane of K562 cells thinned, the boundary was blurred and the fluorescence intensity was significantly reduced after AMP-17 treatment (Figure 2A,B), while the membrane morphology of untreated K562 cells showed a more intact membrane structure. Subsequently, we further determined the damage of cell membrane using fluorescein diacetate (FDA) and oxazolyl yellow YO-PRO-1 (YP-1). It was found that the fluorescence intensity of K562 cells was significantly increased in the AMP-17-treated group (Figure 2C), indicating that the cell membrane was severely damaged. In addition, flow cytometry analysis revealed that the membrane permeability of K562 cells increased by 11.70%, 11.22%, and 26.54% after treatment with different concentrations of AMP-17, respectively. The permeability of cells treated with Ara-C increased by only 3.65% (Figure 2D).

**Figure 3:**
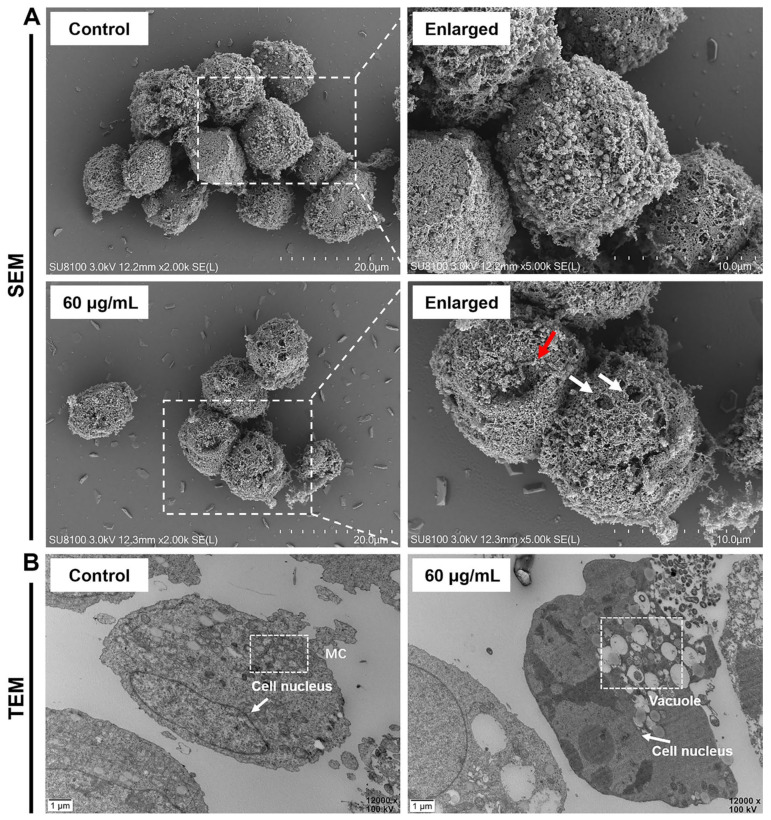
Effect of AMP-17 on the morphology and structure of K562 cells. (**A**) Scanning electron microscopy (SEM) images of K562 cells untreated or treated with 60 μg/mL AMP-17 for 24 h. Images were captured using a 2000× power field. Scale bar, 20 μm. Enlarged images were captured using a 5000× power field. Scale bar, 10 μm. (**B**) Transmission electron microscopy (TEM) images of K562 cells untreated or treated with 60 μg/mL AMP-17 for 24 h. Images were captured using a 12,000× power field. Scale bar, 1.0 μm.

Next, we observed the cell morphology by using scanning electron microscopy (SEM) and transmission electron microscopy (TEM). SEM images showed that the membrane structure of K562 cells was disrupted after treatment with AMP-17, and depressions as well as pore-like structures appeared on the cell surface (Figure 3A), indicating that AMP-17 has a disruptive effect on the cell membrane. TEM images further revealed that cell membrane rupture, leakage of intracellular contents, and the appearance of vacuolated vesicles in the cytoplasm were observed in AMP-17-treated K562 cells (Figure 3B). The untreated cells were structurally intact, intracellularly rich in mitochondria and consistent with typical tumor cell characteristics. These results suggest that the antimicrobial peptide AMP-17 may exert its antitumor effect by disrupting the cell membrane of K562 cells.

### 2.3. AMP-17 Induces Apoptosis in K562 Cells

**Figure 4:**
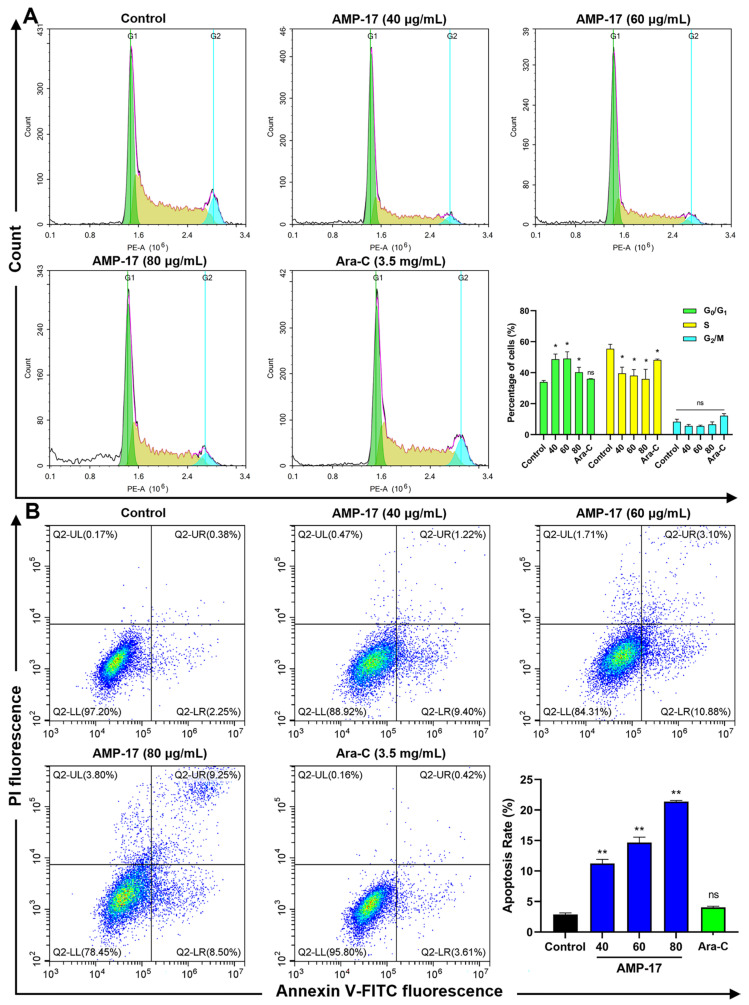
AMP-17 induced apoptosis and cell cycle arrest in K562. (**A**) Cell cycle distribution of AMP-17-treated K562 cells for 24 h was analyzed by flow cytometry. Ara-C was used as a drug control. Data are presented as the mean ± SD of twice independent experiments (* *p* < 0.05). (**B**) Apoptosis rate of K562 cells treated with AMP-17. K562 cells were treated with AMP-17 and Ara-C for 24 h and then determined by Annexin V-FITC/PI assay. Data are presented as the mean ± SD (** *p* < 0.01). The experiment was repeated three independently.

Cell cycle and apoptosis are closely associated in cancer and some anticancer drugs are able to induce apoptosis and cell cycle arrest [24,25]. Thus, we explored the effects of AMP-17 on cell cycle and apoptosis in K562 cells. After AMP-17 treatment, cells were significantly arrested in the G0/G1 phase, and the cell ratio in the S phase decreased with increasing AMP-17 concentration, while there was no significant change in the number of cells in the G2/M phase (Figure 4A). Meanwhile, a similar phenomenon was observed for the positive control drug Ara-C. Ara-C, a pyrimidine class of antimetabolic chemotherapeutic drug, has cell cycle specificity and is more sensitive to cell S phase, i.e., the DNA synthesis phase. Figure 4B shows the percentage of Annexin V-FITC/PI double staining leading to apoptosis. The percentages of apoptotic cells induced with different concentrations of AMP-17 were 11.23%, 14.66%, and 21.39%, respectively, compared to the untreated group (2.90%), while the apoptosis rate of Ara-C-treated cells did not change significantly (4.04%). These data suggest that the anti-cancer mechanism of AMP-17 may involve inhibition of the cell cycle and induction of apoptosis.

**Figure 5:**
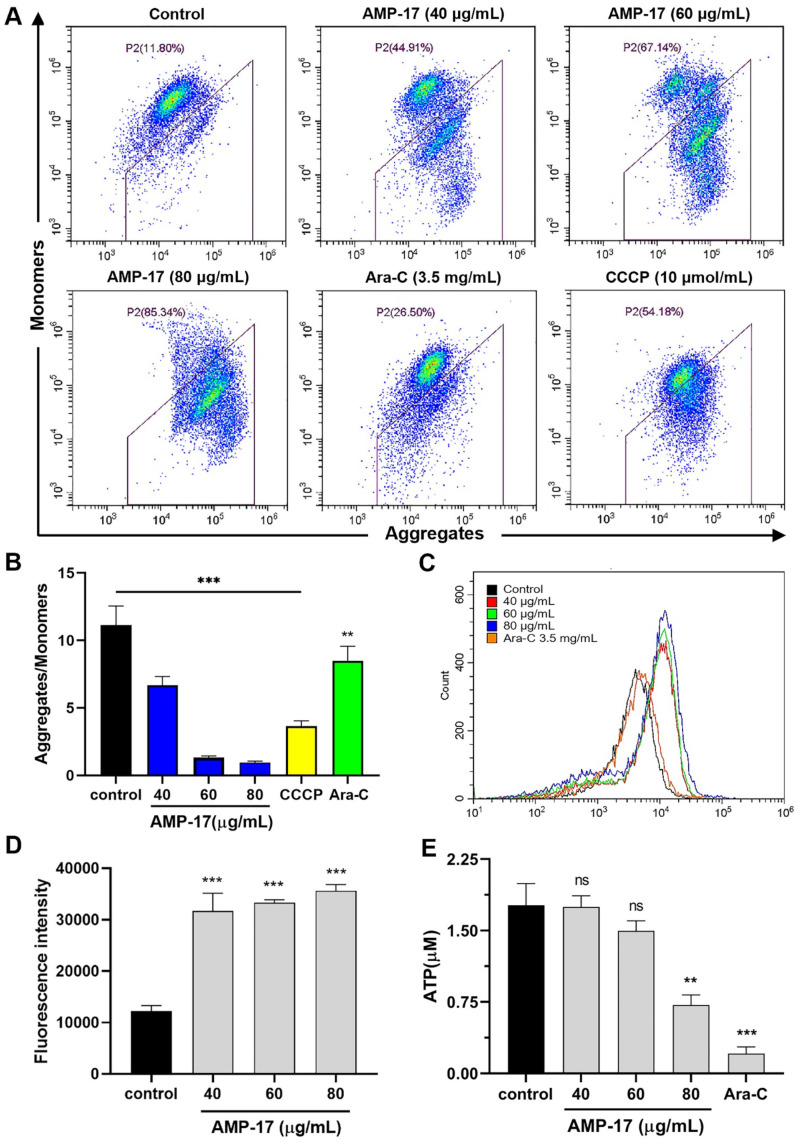
AMP-17-induced apoptosis may be achieved through ROS accumulation, calcium ion release and mitochondrial dysfunction. (**A**) Mitochondrial membrane potential (MMP) changes in K562 cells after 24 h of AMP-17 treatment were detected by using JC-1 staining. CCCP and Ara-C were used as positive control and drug control, respectively. (**B**) Statistical results of the ratio of aggregates to monomer fluorescence intensity of MMP (** *p* < 0.01, *** p < 0.001). The experiment was independently repeated three times. (**C**) Calcium ion levels in the intracellular of K562 cells were analyzed by flow cytometry. K562 cells treated with AMP-17 (40, 60 and 80 μg/mL) were analyzed for Ca^2+^ levels in the intracellular using Fluo-4-AM. The experiment was repeated twice independently. (**D**) Intracellular ROS levels in K562 cells treated with different concentrations of AMP-17 (40, 60 and 80 μg/mL) were measured by using DCFH-DA staining (** *p* < 0.01, *** *p* < 0.001). The experiment was repeated twice independently. (**E**) Changes in intracellular ATP after 24 h treatment of K562 cells with different concentrations of AMP-17 (40, 60, and 80 μg/mL) or Ara-C (3.5 mg/mL) were detected by ATP luciferase. Data are presented as the mean ± SD, and asterisks are indicated as ** *p* < 0.01, *** *p* < 0.001, ns: not significant. The experiment was repeated three times independently.

Mitochondria are the main site of energy metabolism in cells and provide large amounts of ATP to maintain normal physiological functions [26]. Moreover, alteration of mitochondrial membrane potential is one of the main events that cause apoptosis [27]. Therefore, we detected the changes of mitochondrial membrane potential by using the JC-1 probe. As shown in Figure 5A,B, the level of mitochondrial membrane potential (MMP) was significantly reduced in K562 cells after treatment with AMP-17 compared to the untreated. Meanwhile, the MMP levels of both positive control CCCP (Carbonyl cyanide-m-chlorophenylhydrazone) and Ara-C were significantly decreased. Notably, the decreased MMP levels of both Ara-C and CCCP were lower than those of the antimicrobial peptide AMP-17.

It has been shown that Ca^2+^ release and ROS accumulation usually trigger apoptosis [28]. Thus, we measured the changes in calcium ion concentration and ROS levels in K562 cells using Fluo-4/AM and DCFH-DA probes, respectively. AMP-17 treatment resulted in a significant and dose-dependent increase in intracellular Ca^2+^ concentration compared to untreated (Figure 5C). Furthermore, after K562 cells were treated with different concentrations of AMP-17, we observed a significant increase in ROS levels in all groups of cells (Figure 5D). These results suggest that AMP-17 accelerates calcium ion release and ROS accumulation in K562 cells.

Next, we measured the effect of AMP-17 on the ATP content of K562 cells using luciferase. As shown in Figure 5E, 40 μg/mL and 60 μg/mL AMP-17 did not significantly affect the ATP content of K562 cells compared with the untreated group, whereas 80 μg/mL of AMP-17 significantly decreased the ATP content of the cells. Meanwhile, the ATP content of K562 cells was also significantly reduced by Ara-C treatment, and it resulted in a higher reduction in ATP content than the antimicrobial peptide AMP-17. Therefore, the above results suggest that AMP-17-induced apoptosis is closely associated with ROS accumulation, Ca^2+^ elevation, and mitochondrial dysfunction.

### 2.4. AMP-17-Induced Apoptosis Is Caspase-3 and ATP1B1 Dependent

**Figure 6:**
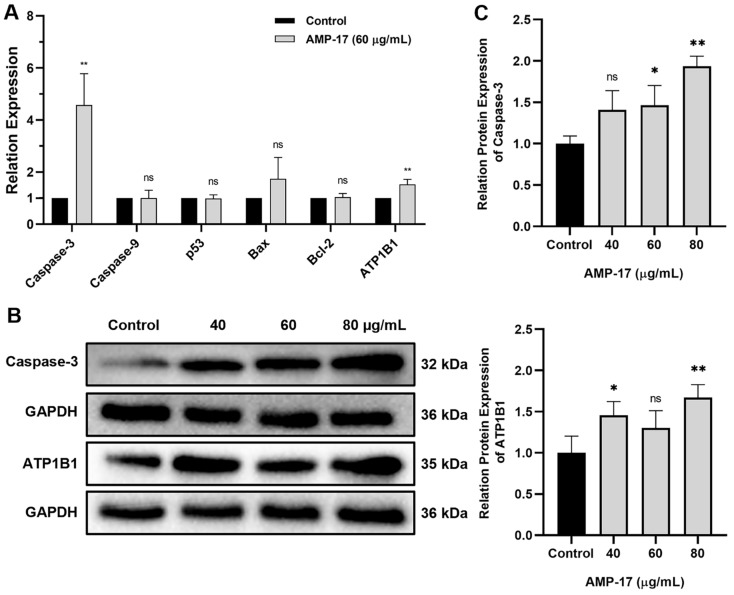
AMP-17-induced apoptosis is Caspase-3 and ATP1B1 dependent. (**A**) After treatment of K562 cells with AMP-17 for 24 h, RNA was extracted and analyzed for mRNA expression of genes associated with apoptosis using qRT-PCR. GAPDH was used as a reference. The experiment was performed in triplicate and repeated three times independently. * *p* < 0.05, ** *p* < 0.01, ns: not significant. (**B**) Expression of Caspase-3 and ATP1B1 proteins were analyzed by using Western blotting after 24 h of treatment of K562 cells with AMP-17. GAPDH was used as a control. The blot represents representative image of three independent replicates. (**C**) The gray value of Caspase-3 and ATP1B1 proteins were analyzed using ImageJ Software (National Institutes of Healthcare, Bethesda, MD, USA). * *p* < 0.05, ** *p* < 0.01, ns: not significant.

The mechanisms of apoptosis are highly complex and sophisticated, involving an energy-dependent cascade of responses to various molecular events. Until now, it has been shown that there are two main apoptotic pathways: the extrinsic or death receptor pathway and the intrinsic or mitochondrial pathway [29]. Based on the results related to AMP-17 disruption of cancer cell membranes and induction of apoptosis, we analyzed the expression of related genes and proteins. Compared with untreated cells, AMP-17-treated cells showed high gene expression, in which the apoptotic gene *Caspase-3* was upregulated 4.60-fold and the cellular transmembrane transporter gene *ATP1B1* was upregulated 1.52-fold, while the expression of *Caspase-9*, *p53*, *Bax*, and *Bcl2* genes was not significantly changed (Figure 6A). Furthermore, treatment of K562 cells with AMP-17 resulted in high expression of the apoptotic protein Caspase-3 and the transmembrane protein ATP1B1 at the protein level (Figure 6B,C). These results suggest that AMP-17 may induce apoptosis by regulating the expression of ATP1B1 and Caspase-3.

## 3. Discussion

Leukemia, as a common malignant disease among children and young people, has been listed as one of the ten priority malignancies for prevention and treatment [30]. Although substantial progress has been made in curing leukemia in recent years, most chemotherapeutic drugs still produce serious toxic side effects on the organism or even drug resistance. Therefore, the emergence of antimicrobial peptides has provided a novel strategy for cancer treatment. Compared with conventional antitumor drugs, antimicrobial peptides exhibit significant advantages, such as higher specificity, cell-selective toxicity, and lower drug resistance [31]. In this study, we provided a novel antimicrobial peptide, AMP-17, which exhibited potent antitumor activity against K562 cells. Furthermore, our results initially elucidate that the anti-proliferative effect of AMP-17 may be achieved through membrane disruption as well as mitochondria-mediated apoptosis.

Membranolytic has been suggested as a target for the anticancer mechanism of AMPs [32,33]. AMPs serve as anticancer peptides (ACPs) that have the ability to cross cell membranes and kill cancer cells. It has been shown that the specific recognition of tumor cells is caused by the presence of negatively charged phosphatidylserines (PS) on their surface, which results from high levels of ROS and hypoxia, and that these ROS and hypoxia alter the tumor microenvironment and induce dysregulation of membrane phospholipids [10,34]. Furthermore, surface changes in tumor cell membranes are not limited to phospholipid and protein content. The glycosylation pattern of proteins and polar phospholipid heads may also be altered during tumor development [33]. Indeed, tumor cells lose the asymmetry of phospholipid distribution between the outer and inner layers of the plasma membrane, exposing PS to the outer layer of tumor cells [35]. Thus, AMPs are able to preferentially bind and insert into negatively charged cell membranes, specifically selectively inhibiting cancer cells by electrostatic attraction [36]. Similarly, AMP-17 exerts a membrane-disrupting effect on cancer cells. In membrane-associated experiments, we observed that K562 cell membranes were damaged after treatment with AMP-17 and pore shapes were formed in the membranes. In conclusion, AMP-17 exerts an action on cell membranes and alters the permeability of cancer cells.

The anticancer effects of AMPs can follow different pathways, including ROS accumulation, mitochondrial membrane potential depolarization, induction of apoptosis, etc. [18,19]. It has been shown that an increase in cytoplasmic Ca^2+^ can act as an apoptotic signal to initiate apoptosis [37]. Furthermore, intracellular Ca transients have been implicated in cell cycle regulation and cell proliferation [38]. Thus, necrotic cell death was early associated with intracellular Ca^2+^ overload and caused altered mitochondrial membrane permeability and dysfunction [37,38]. AMP penetrates the cytoplasm and enters the mitochondria, damaging the mitochondrial membrane and inducing excessive accumulation of ROS, disrupting membrane potential, and inducing apoptosis [27,39]. Meanwhile, disruption of mitochondria leads to impairment of the respiratory chain and disruption of energy metabolism, further inducing apoptosis [39,40]. In the present study, our results showed that AMP-17 induced apoptosis in K562 and that the induced apoptosis was highly correlated with increased Ca^2+^ inward flow, ROS accumulation, decreased Δψm and decreased ATP content.

It has been reported that caspases are key enzymes in the molecular mechanism of apoptosis and its mediated apoptosis is triggered through both extrinsic/receptor and intrinsic/mitochondrial pathways [41]. In addition, activation of caspases appears to cause irreversible induction of programmed cell death. Until now, ten major caspases have been identified, of which caspase-9 is an initiator of apoptosis and Caspase-3 is an effector or executor [29]. In this study, we found that AMP-17-induced apoptosis activated only the Caspase-3-mediated mitochondrial pathway, but not the Bcl-2 and p53 pathways, suggesting that AMP-17-induced apoptosis may be dependent on the Caspase-3-mediated mitochondrial pathway. Our results confirm the study of Wang et al. [42] that ropivacaine is involved in mitochondrial and Caspase-dependent apoptotic pathways. Na^+^, K^+^-ATPase is a transmembrane protein that regulates and maintains the Na^+^ and K^+^ gradients required for cellular homeostasis [43]. The enzyme is composed of a catalytic α subunit, a regulatory β subunit and an optional γ (FXYD2) subunit, and pumps sodium ions out and potassium ions into the cell at the expense of ATP thereby generating a sodium gradient across the plasma membrane [44,45]. Our results revealed that AMP-17 treatment resulted in upregulation of *ATP1B1* gene expression, which further increased the expression of the transmembrane protein ATP1B1 and decreased the ATP content. Therefore, we speculated that ATP1B1 expression may cause sodium ions to be pumped out and potassium ions to enter intracellularly at the cost of ATP consumption, thereby creating a sodium gradient across the plasma membrane and contributing to an imbalance in intra- and extracellular ion concentrations as well as altered osmotic pressure, which subsequently, further triggered Caspase-3 activity to induce apoptosis.

**Figure 7:**
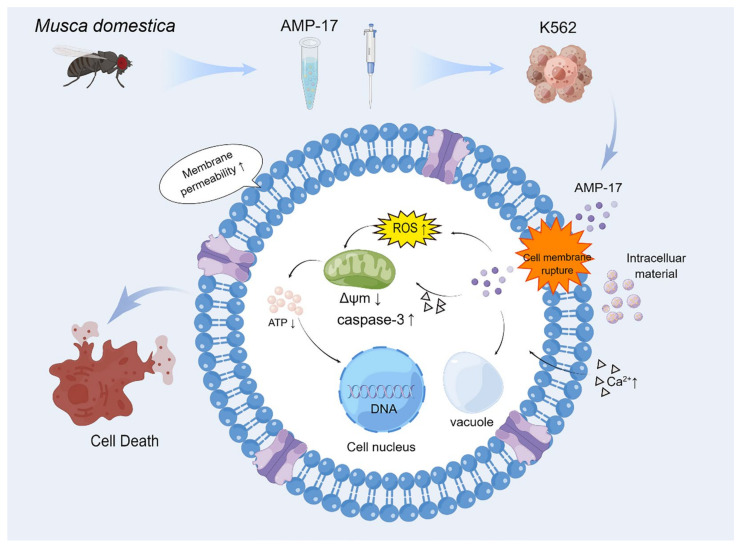
Schematic diagram of the proposed mechanism of AMP-17 against K562 cells. AMP-17 disrupts K562 cell membrane integrity and increases membrane permeability, allowing the peptide to enter the intracellular. Subsequently, this peptide stimulation resulted in accumulation of ROS and increased inward flow of Ca^2+^, which further caused depolarization of mitochondrial membrane potential and impaired ATP synthesis. Therefore, AMP-17 may induce apoptosis in leukemia K562 cells through disrupting mitochondria and activating Caspase-3 activity. By Figdraw (www.figdraw.com, accessed on 11 May 2022).

In conclusion, this study demonstrates that AMP-17 interacts with cell membranes to disrupt membrane integrity and increase membrane permeability of tumor cells. Furthermore, AMP-17-induced apoptosis may be closely related to increased calcium influx, ROS accumulation, and mitochondrial dysfunction (Figure 7). Thus, AMP-17 may induce apoptosis in leukemia K562 cells by disrupting mitochondria and activating Caspase-3 activity. Overall, this finding may provide some experimental basis for the interaction between AMPs and cancer cells. However, this study was limited to in vitro experiments and further in vivo validation is required.

## 4. Material and Methods

### 4.1. Antimicrobial Peptide

The recombinant protein AMP-17 was prepared according to the previous method [21,23]. Briefly, a single positive colony of *E. coli* BL21 (DE3) containing plasmid pET-28a (+)—(AMP-17) was inoculated in LB liquid medium containing kanamycin and grown overnight by shaking. The following day, the above bacterial suspension was expanded at a ratio of 1:100 to logarithmic growth phase. Then, Isopropyl-beta-D-thiogalactopyranoside (IPTG, Solarbio, Beijing, China) was added to the bacterial suspension and grown at 32 °C for 24 h. Subsequently, the recombinant protein AMP-17 was obtained by ultrasonic fragmentation, inclusion body lysis, Ni-NTA column purification, and ultrafiltration.

### 4.2. Cell Culture

Human chronic myeloid leukemia cells K562 was purchased from Procell Life Science &Technology Co., Ltd. (Wuhan, China). Cells were cultured in RPMI 1640 medium (Gibco, Grand Island, NY, USA) containing 10% fetal bovine serum (FBS, Gibco, USA), 100 U/mL penicillin (Gibco, USA), and 100 µg/mL streptomycin (Gibco, USA) and maintained at 37 °C in a humidified 5% CO_2_ incubator.

### 4.3. Cell Proliferation and Viability Assays

The anticancer activity of AMP-17 was determined according to the protocol of the Cell Counting Kit-8 (CCK-8, APE-BIO, Houston, Texas, USA). Briefly, 100 μL of K562 cells at a concentration of 2 × 10^4^ cells/mL were inoculated in 96-well plates and incubated at 37 °C in a 5% CO_2_ incubator for 24 h. Then, cells were treated with different concentrations of AMP-17 (0, 15, 30, 60, 120, 240 μg/mL) for 24 h and incubated with CCK-8 for 2 h in the dark. The absorbance of each well was measured at OD_450_ using an iMark^TM^ microplate reader (Bio-Rad, Hercules, CA, USA), and the growth inhibition rate and half inhibitory concentration (IC_50_) of the cells were calculated. For time-growth curve assay. A total of 100 μL of AMP-17 (40, 60, and 80 μg/mL) was added to 96-well plates containing K562 cells and incubated at 37 °C for 24, 48, and 72 h, respectively. Subsequently, cells from each time point (24, 48, 72 h) were transferred to new 96-well plates and 10 μL of CCK-8 was added and incubated in the dark for 2 h. The absorbance of each well was measured at OD_450_ using an iMark^TM^ microplate reader (Bio-Rad, USA), and the time-growth curve was plotted.

### 4.4. Trypan Blue Staining

Trypan Blue staining assay was performed as described by Crowley et al. [46], with minor modifications. Briefly, 1 mL of K562 cells at a density of 1 × 10^6^ cells/mL were inoculated in 24-well plates and incubated at 37 °C in a 5% CO_2_ incubator for 24 h. Then, AMP-17 (40, 60, and 80 μg/mL) was added to the cells for 24 h. After incubation, the cells were stained with Trypan Blue (Solarbio, China) and the cells were observed by using an inverted light microscope (Olympus CKX31, Tokyo, Japan).

### 4.5. Cell Cycle Analysis

Cell cycle assay was performed according to the Cell Cycle Kit (Beyotime, Jiangsu, China) protocol. Briefly, K562 cells were seeded at a density of 1 × 10^6^ cells/mL in 24-well plates and maintained for 24 h. Cells were then treated with AMP-17 (40, 60, 80 μg/mL) and 3.5 mg/mL Cytosine arabinoside (Ara-C, Yuanye-Bio, Shanghai, China) for 24 h, respectively. After incubation, cells were collected, washed twice with phosphate-buffered solution (PBS, Solarbio, China), and fixed with 70% ethanol at 4 °C overnight. Subsequently, 50 μg/mL propidium iodide (PI) was added to each well and incubated for 30 min at 37 °C in the dark. Cells were analyzed using a flow cytometer (ACEA NovoCyte, San Diego, CA, USA).

### 4.6. Apoptosis Analysis

Apoptosis was determined according to the Apoptosis Kit (Beyotime, China) protocol. Briefly, K562 cells at a density of 1 × 10^5^ cells/mL were inoculated in 24-well plates and maintained for 24 h. Then, the cells were treated with AMP-17 (40, 60, and 80 μg/mL) or Ara-C (3.5 mg/mL) for 24 h. After incubation, the cells were collected and washed twice with PBS. Subsequently, cells were resuspended by adding Annexin-V FITC binding solution and incubated with Annexin-V FITC and PI staining solution for 20 min at room temperature in the dark. The stained cells were analyzed using a flow cytometer (Beckman, Brea, CA, USA).

### 4.7. Membrane Permeability Assay

K562 cells at a density of 1 × 10^6^ cells/mL were seeded into 24-well plates and maintained for 24 h. Then, the cells were treated with different concentrations of AMP-17 (40, 60, 80 μg/mL) or Ara-C (3.5 mg/mL) for 24 h. After incubation, the cells were collected and washed twice with PBS. Subsequently, membrane permeability assays were performed according to the manufacturer’s instructions of fluorescein diacetate (FDA, Yuanye-Bio, China) and oxazolyl yellow YO-PRO-1/PI kits (YP-1/PI, Beyotime, China), respectively, and stained cells were analyzed using a SYNERGY-H4 multifunctional fluorescence microplate reader (Bio-Tek, Winooski, VM, USA) and a flow cytometer (Beckman, USA), respectively.

### 4.8. Confocal Laser Scanning Microscope

The effect of AMP-17 on K562 cell membranes was observed according to the Cell Membrane Fluorescent Probe DiI Reagent Merchant protocol. Briefly, K562 cells (1 × 10^6^ cells/mL) were inoculated into 24-well plates and maintained for 24 h. Cells were then treated with different concentrations of AMP-17 (40, 60, and 80 μg/mL) for 24 h. Cells were collected and fixed with 4% paraformaldehyde for 10 min. Subsequently, cells were washed three times with PBS, stained with a final concentration of 10 μM 1,1′-Dioctadecyl-3,3,3′,3′-tetramethylindocarbocyanineperchlora-te (Dil, Yuanye-Bio, China) and incubated for 20 min, and sealed with anti-fluorescence quencher containing 4,6-Diamidino-2-phenylindole (DAPI, Solarbio, China). Samples were observed using a confocal laser scanning microscope (CLSM, Olympus FV1000, Japan).

### 4.9. Scanning Electron Microscope

The assay was performed as described by Liang et al. [47], with slight modifications. Briefly, K562 cells at a density of 1 × 10^6^ cells/mL were inoculated into 6-well plates and maintained for 24 h. Then, the cells were treated with 60 μg/mL of AMP-17 for 24 h. After incubation, the cells were collected, washed with PBS, and fixed with 2.5% glutaraldehyde at 4 °C for 2 h. Subsequently, gradient dehydration was performed with different concentrations of ethanol. Samples were vacuum dried and then gold-sprayed with an ion sputterer and observed using a scanning electron microscope (SEM, Hitachi, SU 8100, Japan).

### 4.10. Transmission Electron Microscope

The assay was performed as described by Ju et al. [14], with slight modifications. Briefly, K562 cells (1 × 10^6^ cells/mL) were inoculated into 6-well plates and maintained for 24 h. Then, the cells were treated with 60 μg/mL of AMP-17 for 24 h. After incubation, the cells were collected and washed with PBS. Then, the samples were washed, fixed with 1% osmium tetroxide for 1 h, and dehydrated with a series of graded ethanol for 10 min. Finally, the cells were embedded, ultra-thin sections were prepared with an ultrathin microtome, and the samples were observed using a transmission electron microscope (TEM, JEOL, JEM-1011, Tokyo, Japan).

### 4.11. ROS Assay

In brief, K562 cells at a density of 1 × 106 cells/mL were seeded into 24-well plates and maintained for 24 h. Then, cells were treated with different concentrations of AMP-17 (40, 60, 80 μg/mL) for 12 h. After incubation, cells were collected and washed twice with PBS. Subsequently, 2,7-Dichlorodihydrofluorescein diacetate (DCFH-DA, Yuanye Bio, China) probes were added at a ratio of 1: 1000 and incubated for 20 min at 37 °C in the dark. The stained cells were analyzed using flow cytometry (Beckman, USA).

### 4.12. Detection of Intracellular Ca^2+^ Concentration

Intracellular Ca^2+^ concentration was determined according to the Fluo-4, AM kit protocol. Briefly, K562 cells (1 × 10^6^ cells/mL) were inoculated into 24-well plates and maintained for 24 h. Then, the cells were treated with AMP-17 (40, 60, and 80 μg/mL) or Ara-C (3.5 mg/mL) for 24 h. After incubation, the cells were collected and washed twice with PBS. Subsequently, 1-[2-Amino-5-(2,7-difluoro-6-hydroxy-3-oxo-9-xanthenyl)phenoxy]-2-(2-amino-5-methylphenoxy)ethane-N,N,N′,N′-tetraacetic acid, pentaacetox-ymethyl ester (Fluo-4/AM, Solarbio, China) working solution was added to each group of cells and incubated in the dark for 20 min at 37 °C. After that, 5 times the volume of HBSS (Solarbio, China) buffer containing 1% FBS was added to each group of cells and incubated for 40 min. Finally, the cells were washed three times with HEPES buffer saline (Solarbio, China) and resuspended, incubated at 37 °C for 10 min, and then the Ca^2+^ concentration was measured by flow cytometry (Beckman, USA).

### 4.13. Mitochondrial Membrane Potential Assay

K562 cells (1 × 10^6^ cells/mL) were inoculated into 24-well plates and maintained for 24 h. Then, cells were treated with AMP-17 (40, 60, 80 μg/mL) and Ara-C (3.5 mg/mL) for 24 h, respectively. After incubation, cells were collected and washed twice with PBS. Each group of cells was added with 0.5 mL of JC-1 staining solution according to the Mitochondrial Membrane Potential Kit (Beyotime, China) protocol and incubated in the dark for 20 min at 37 °C. The samples were measured using a flow cytometry (Beckman, USA).

### 4.14. Detection of ATP Content

K562 cells (1 × 10^6^ cells/mL) were inoculated into 24-well plates and maintained for 24 h. Then, the cells were treated with AMP-17 (40, 60, 80 μg/mL) and Ara-C (3.5 mg/mL) for 24 h, respectively. After incubation, the cells were collected and lysed by adding 200 μL of lysis solution to each well. Subsequently, the lysed cells were centrifuged at 4 °C for 5 min at 12,000× *g*. A total of 20 μL of supernatant from each group of cells was added to a 96-well plate containing 100 μL of adenosine 5’-triphosphate (ATP, Beyotime, China) working solution and detected using a multifunctional fluorescence microplate reader (Bio-Tek, USA).

### 4.15. Real-Time PCR Analysis

Cell culture was performed as described above. K562 cells were treated with 60 μg/mL of AMP-17 for 24 h. After incubation, total RNA was extracted according to the RNA Rapid Extraction Kit (Yishan, China) extraction protocol, and then reverse transcribed into cDNAs using the M16325 RevertAid First Strand cDNA Synthesis Kit (Thermo, USA). Next, quantitative real-time polymerase chain reaction assays (qRT-PCR, 7300 Applied Biosystems, USA) were performed using the PowerUpTM SYBRTM Green Master Mix (Thermo, USA) and specific primers (Table 1). The level was calculated by using the 2^−ΔΔCt^ method, and GAPDH was served as the internal control.

### 4.16. Western Blotting

Cell culture was performed as described above. K62 cells were treated with AMP-17 (40, 60, and 80 μg/mL) for 24 h. After incubation, cells were collected and lysed with RIPA lysis buffer for 30 min, and protein concentrations were detected using the BCA Protein Assay Kit (Beyotime, China). Next, equal amounts of protein samples were denatured and separated by gel electrophoresis using 12.5% SDS-PAGE (Meilunbio, Dalian, China) and transferred to PVDF membranes. Subsequently, the membranes were incubated in the blocking solution for 10 min and then co-incubated overnight with primary antibodies (ATP1B1, Caspase-3 and GAPDH). The following day, the membranes were incubated with goat anti-rabbit secondary antibody (Thermo, Waltham, MA, USA, Cat No: C31460100,) at room temperature for 2 h. The antibody (ATP1B1, Cat No: GR323222-7) was purchased from Abcam (Cambridge, UK) and the antibodies (Caspase-3, Cat No: 19677-1-AP and GAPDH, Cat No: 10494-1-AP) were purchased from Proteintech (Waltham, MA, USA). The blot images were obtained by a Bio-Rad Image Lab^TM^ Software System (Bio-Rad, USA) and analyzed using ImageJ Software (National Institutes of Healthcare, USA).

### 4.17. Statistical Analysis

Statistical analysis was performed using GraphPad Prism 8.0.1 software (GraphPad Software, San Diego, CA, USA). Data were expressed as mean ± SD and analyzed by Student’s t-test and one-way ANOVA, and *p* < 0.05 was considered statistically significant.

## Figures and Tables

**Table 1:** Oligonucleotide primers used in this study.

Gene Name		Primer Sequences
*Caspase-3*	FORWARD	5′-GCAGCAAACCTAGGGAAAC-3′
	REVERSE	5′-TGTCGGCATACTGTTTCAGCA-3′
*Caspase-9*	FORWARD	5′-TGTGGTGGTCATCCTCTCTCA-3′
	REVERSE	5′-GTCACTGGGGGTAGGCAAACT-3′
*Bax*	FORWARD	5′ -CATCTCCTTGCTCGTAGTCTAGAGC-3′
	REVERSE	5′ -CATTGTGATGGACTCCGGAGACGG-3′
*Bcl-2*	FORWARD	5′-TTTGAGTTCGGTGGGGTCAG-3′
	REVERSE	5′-TGACTTCACTTGTGGCCCAG-3′
*P53*	FORWARD	5′-GGGTTAGTTTACAATCAGCCACATT-3′
	REVERSE	5′-GGCCTTGAAGTTAGAGAAAATTCA-3′
*ATP1B1*	FORWARD	5′-GCCAGGATTAACACAGATTCCTCAG-3′
	REVERSE	5′-CCTTATCTTCATCTCGCTTGCC-3′
*GAPDH*	FORWARD	5′-ACACCCACTCCTCCACCTTT-3′
	REVERSE	5′-TAGCCAAATTCGTTGTCATACC-3′

## Data Availability

The data presented in this study, are available in this article.
